# Philadelphia chromosome-positive de novo myelodysplastic syndrome with the p230 *BCR::ABL1* fusion gene: a case report

**DOI:** 10.1007/s12185-025-04083-0

**Published:** 2025-10-28

**Authors:** Hidetsugu Kawai, Hidehito Fukushima, Yasuhito Nannya, Makoto Onizuka, Yoshiaki Ogawa, Hiroshi Kawada

**Affiliations:** 1 Department of Hematology/Oncology, Tokai University School of Medicine, 143 Shimokasuya, Isehara, Kanagawa Japan; 2 Department of Hematology, Hiratsuka Mutual Aid Hospital, Hiratsuka, Kanagawa 259-1193 Japan; 3 Division of Hematopoietic Disease Control, Institute of Medical Science, The University of Tokyo, Tokyo, Japan

**Keywords:** Philadelphia chromosome-positive de novo myelodysplastic syndromes, Micro *BCR::ABL1*, Tyrosine kinase inhibitor

## Abstract

De novo Ph-positive MDS with the micro *BCR::ABL1* (e19a2/p230) transcript is rare. Here, we report a case of MDS with multilineage dysplasia in an 86-year-old woman. Reverse transcription polymerase chain reaction (RT-PCR) showed the following karyotype: 46, XX, t(9;22)(q34.1;q11.2), i(17)(q10) [20], and p230 *BCR::ABL1*. Targeted NGS at diagnosis revealed mutations in *CSF3R*, *BCOR*, *SRSF2*, and *ASXL1*. Low-dose dasatinib (20 mg/day) reduced *BCR::ABL1* levels (FISH negative, RT-PCR positive), but had no effect on anemia, dysplasia, or transfusion frequency. At six months, panel results showed loss of all mutations except *ASXL1*, as well as clearance of Ph-positive subclones. However, an *ASXL1* founder clone persisted. Post-treatment chromosome analysis was not feasible because of poor cell growth. Serial genomics suggested that Ph positivity was a late/secondary event on a pre-existing *ASXL1*-mutant background. In Ph-positive de novo MDS, *BCR::ABL1* transcript responses alone may not reflect disease control when an adverse founder clone persists. Integrating panel-based NGS with fusion transcript monitoring may improve therapeutic decision-making and prognostic assessment.

## Introduction

Ph-positive de novo myelodysplastic syndrome has rarely reported [1, 2], and its clinicopathologic features and management remain insufficiently defined. Among *BCR::ABL1* variants, micro *BCR::ABL1* (μ-bcr) is the fusion of *BCR* exon 19 and *ABL1* exon 2 (e19a2), which results in a 230-kDa protein, and can be a molecular diagnostic marker for neutrophilic-chronic myeloid leukemia (CML-N) [3]. The significance of u-bcr in Ph-positive de novo MDS is poorly characterized owing to the paucity of reports.

Two literature reviews (Ma J et al. and Qi S et al.) identified 24 unique cases after accounting for seven overlapping reports, and only one involved the μ-bcr [4, 5]. Against this background, we herein report a case of Ph-positive de novo MDS with μ-bcr and isochromosome 17q [i(17q)], documenting serial genomic changes during low-dose dasatinib.

## Materials and methods

Total RNA was extracted from bone marrow cells, and complementary DNA (cDNA) was synthesized in vitro for RT-PCR to detect *BCR::ABL1* transcripts, including μ-bcr e19a2 (p230). NGS analysis was performed using the Tru Sight Myeloid Sequencing Panel (Illumina). Genomic DNA was extracted from bone-marrow cells at diagnosis and after 6 months of dasatinib for targeted NGS using a myeloid panel per validated laboratory procedures. The analytical limit of detection for single-nucleotide variants on this panel was approximately 2–5% variant allele frequency, depending on coverage.

Quality filtering for the generated read data was performed using fastp (ver. 0.23.2) with default parameters. The filtered sequence data were aligned to the RefSeq Human Genome GRCh37 using Burrows-Wheeler Aligner (ver. 0.7.12). The mapping data were processed using Samtools (ver. 1.12), Picard (ver. 2.23.0), and GATK (ver. 4.1.9.0). Calling of single-nucleotide variants (SNVs) was performed using Mutect2, HaplotypeCaller, VarScan2 (ver. 2.3), LoFreq (ver. 2.1.3.1), and FreeBayes (ver. 1.3.6,arXiv:1207.3907). The identified variants were aligned using the bcftools (ver. 1.18) and annotated using ANNOVAR.

## Case presentation

**Fig. 1:**
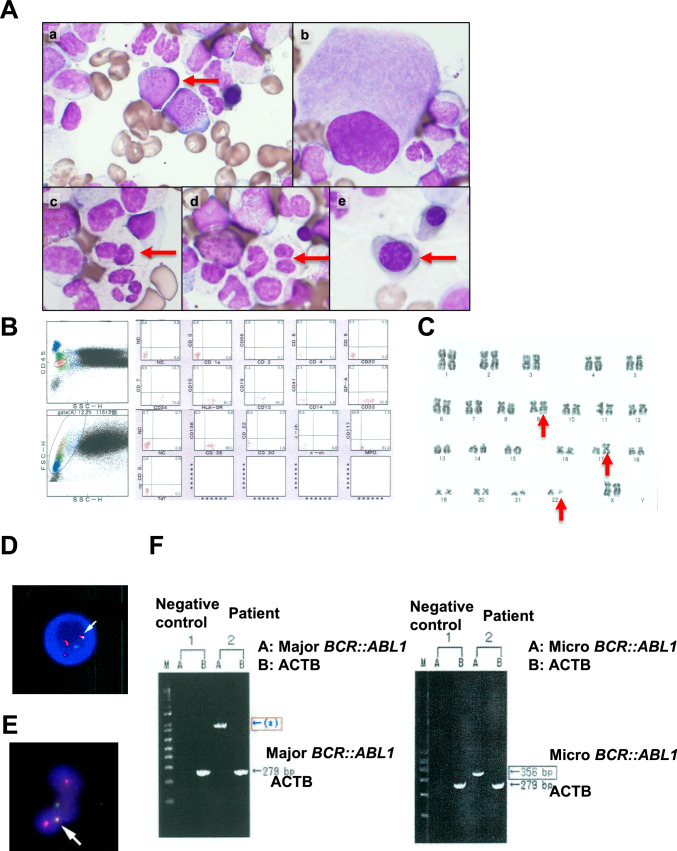
Bone marrow sample and detection of the *BCR::ABL1* fusion gene at the diagnosis. **A** Blasts and multilineage dysplasia of the bone marrow sample obtained at the diagnosis. **B** Flow cytometry by CD45 gating. **C** Chromosomal analysis. **D** FISH analysis of *bcr::abl1* in bone marrow. **E** FISH analysis of neutrophil*-bcr::abl1* in peripheral blood. **F** RT-PCR of major *bcr::abl1* (left lane) and micro *bcr::abl1* (right lane). Actin beta (ACTB) was used as the control. The band of the major *bcr::abl1* should be shown in 371 bp, but an abnormal band is exhibited in the position of the asterisk mark (*)

An 86-year-old woman presented with anemia. She had chronic heart failure (CHF) and chronic kidney disease (CKD). Laboratory tests revealed the following findings: hemoglobin, 6.5 g/dL; platelet count, 158 × 10^9^/L; leukocyte count, 5.0 × 10^9^/L without blasts; prothrombin time (PT), 27.1 s; activated partial thromboplastin time (APTT), 50.9 s; FDP, 2.9 µg/mL; D-dimer, 0.80 µg/mL; fibrinogen 291.0 mg/dL. A bone marrow examination revealed a nucleated cell count of 426 × 10^9^/L, myeloblasts with 2.3% positive for peroxidase (Fig. 1A-a), and multilineage dysplasia was recognized including neutrophilic degranulation, pseudo-Pelger nuclear anomaly, erythroblast of megaloblastic change and single nucleus megakaryocyte (Fig. 1A-b–e). Flow cytometry revealed positivity for several myeloid markers including CD 13, CD 33, CD 34, CD 117, HLA-DR and MPO (Fig. 1B). A chromosomal analysis revealed 46, XX, t(9;22)(q34.1;q11.2), i(17)(q10) [20] (Fig. 1C). A FISH analysis of *bcr::abl1* revealed 87% positivity in the bone marrow (Fig. 1D) and peripheral neutrophils (Fig. 1E). Reverse transcription polymerase chain reaction (RT-PCR) results were positive for μ-bcr and negative for M-bcr (Fig. 1F). RT-PCR analysis revealed that μ-bcr mainly produced Ph-positive MDS in this case. The patient was finally diagnosed with myelodysplastic syndrome with multilineage dysplasia (MDS-MLD) with μ-bcr. The revised International Prognostic Scoring System (IPSS-R) score was high (4.5).

**Fig. 2:**
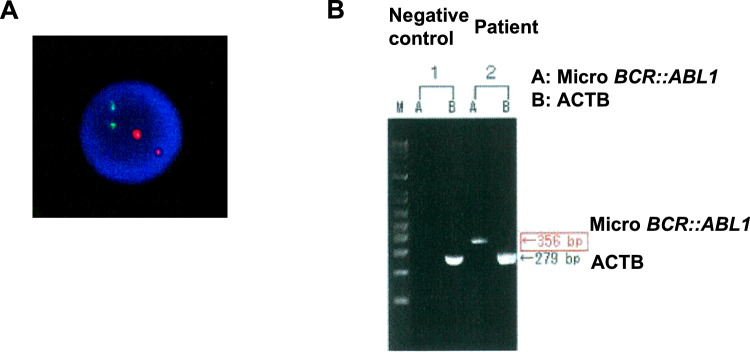
FISH analysis and RT-PCR after dasatinib treatment. **A** FISH analysis of *bcr::abl1* in bone marrow. **B** RT-PCR of micro *bcr::abl1*

The use of dasatinib for MDS has not been approved by the insurance. After the use of dasatinib was approved by the ethics committee, informed consent for dasatinib treatment was obtained. The patient was treated with dasatinib (20 mg/day) and darbepoetin alfa according to age and CKD. After 6 months, a bone marrow examination revealed a nucleated cell count of 99 × 10^9^/L, myeloblasts with 2.3%, and multilineage dysplasia. Chromosomal analysis was not evaluated by poor cell growth. Therefore, it is unclear whether i(17)(q10) remained. FISH analysis of *bcr::abl1* did not detect split signals, but RT-PCR was positive for μ-bcr (Fig. 2). Dasatinib and darbepoetin alfa were concurrently administered, but the frequency of blood transfusion did not change, and bone marrow dysplasia persisted. No adverse effects, including pleural effusion, were observed, and the patient was able to continue treatment with dasatinib 20 mg.

NGS revealed mutations in *CSF3R*, *BCOR*, *SRSF2*, and *ASXL1* at the time of diagnosis; the frequency of each variant allele is shown in Table 1. During the course of treatment, all mutations except for *ASXL1* became undetectable by 6 months. However, 8 months after diagnosis, the patient’s performance status declined due to congestive heart failure and chronic kidney disease, and she became unable to continue outpatient visits. Given that the *ASXL1* mutation–associated with treatment resistance and poor prognosis–persisted despite dasatinib therapy, and her general condition had deteriorated, and the treatment was discontinued.

**Table 1:** NGS analysis at the diagnosis and after dasatinib treatment

Gene	At a diagnosis (VAF)	After dasatinib treatment (VAF)
*BCOR* c.C4870T: p.Q1624X	0.29	0
*CSF3R* c.690delC: p.P230fs	0.1	0
*CSF3R* c.1404delC: p.P468fs	0.071	0
*SRSF2* c.287delC: p.P96fs	0.06	0
*ASXL1* c.1927delG: p.G643fs	0.08	0.1

*NGS* next generation sequencing, *VAF* variant allele frequency

## Discussion

Cases of Ph-positive de novo MDS are rare. Ma J et al. and Qi S et al. summarized 24 cases of Ph-positive de novo MDS in a case report with two literature reviews; however, only one case of μ-bcr could not continue imatinib treatment [4, 5]. We report a case of Ph-positive de novo MDS with μ-bcr in which the patient continued to take dasatinib for 8 months.

After 6 months of low-dose dasatinib, the *BCR::ABL1* clone diminished, while *ASXL1* persisted and marrow dysplasia and transfusion need were unchanged. This pattern supports *ASXL1* as a founder clone and suggests that the Ph chromosome was a secondary event in disease evolution, contrasting with its initiating role in CML or Ph-positive ALL.

Persistence of an adverse-risk founder such as *ASXL1* may predict limited benefit of tyrosine kinase inhibitors (TKIs) monotherapy despite transcript responses. Management in such settings may consider dose escalation when feasible, hypomethylating agent-based therapy, or allogeneic transplantation in eligible patients, alongside serial NGS to align treatment with clonal dynamics [6–8].

In this case, i(17q) is a single chromosomal abnormality. Isolated isochromosome 17q, i(17q), accounts for less than 1% of myeloid neoplasms, including AML, MDS, and myeloproliferative neoplasms (MPN), and frequently harbors mutations in *SETBP1*, *ASXL1*, *SRSF2*, and *NRAS* [9, 10]. The present case of i(17q) featured typical mutations in *SRSF2* and *ASXL1*.

Reported outcomes are heterogeneous: Additional chromosomal abnormalities were frequently found. Out of 24 patients, 9 (36%) cases were Ph-positive without additional chromosomal abnormalities, 9 (36%) cases were Ph-positive with a complex karyotype, and 6 (24%) cases were Ph-positive with single additional chromosomal abnormalities. TKIs sensitivity is sometimes seen when Ph occurs alone or with a single additional lesion, whereas durability is short when complex karyotypes or multiple adverse mutations coexist. Consolidating these observations emphasizes the contrast and may aid clinical decision-making.

Low-dose dasatinib (20 mg/day) reduced μ-bcr levels. If dasatinib was increased to a regular dose and used for a long time, it might have been useful for reducing μ-bcr. It was assumed that the residual *ASXL1* mutation after dasatinib treatment was a founder clone, and that mutations including *CSF3R*, *BCOR* and *SRSF2* were subclones after the acquisition of μ-bcr. Owing to the residual *ASXL1* mutation, dasatinib treatment did not improve anemia. Therefore, dasatinib monotherapy has a limited effect.

In conclusion, we reported a case of Ph-positive de novo MDS with μ-bcr. Mutations in *CSF3R*, *BCOR*, *SRSF2*, *ASXL1,* and isolated isochromosome 17q may contribute to the progression of MDS. Before and after treatment with TKIs, the detection of residual founder clones using NGS is useful for deciding the therapeutic strategies and predicting the prognosis. Further analyses with the accumulation of additional cases are necessary to improve the prognosis.
